# *Citrobacter freundii *infection after acute necrotizing pancreatitis in a patient with a pancreatic pseudocyst: a case report

**DOI:** 10.1186/1752-1947-5-51

**Published:** 2011-02-07

**Authors:** Antonio Lozano-Leon, Jose Iglesias-Canle, Julio Iglesias-Garcia, Jose Larino-Noia, Enrique Dominguez-Muñoz

**Affiliations:** 1 Department of Gastroenterology and Foundation for Research in Digestive Diseases, University Hospital Santiago de Compostela, Spain, A Choupana s/n, 15706. Santiago de Compostela, Spain

## Abstract

**Introduction:**

Infections are the most frequent and severe complications of acute necrotizing pancreatitis with a mortality rate of up to 80 percent. Although experimental and clinical studies suggest that the microbiologic source of pancreatic infection could be enteric, information in this regard is controversial.

**Case presentation:**

We describe a *Citrobacter freundii *isolation by endoscopy ultrasound fine needle aspiration in a 80-year-old Caucasian man with pancreatic pseudocyst after acute necrotizing pancreatitis.

**Conclusion:**

Our case report confirms that this organism can be recovered in patients with a pancreatic pseudocyst. On-site cytology feedback was crucial to the successful outcome of this case as immediate interpretation of the fine needle aspiration sample directed the appropriate cultures and, ultimately, the curative therapy. To the best of our knowledge, this is the first reported case of isolated pancreatic *C. freundii *diagnosed by endoscopy ultrasound fine needle aspiration.

## Introduction

The infection of pancreatic and peripancreatic tissue in the course of severe acute pancreatitis (AP) occurs most frequently in patients with extensive pancreatic necrosis. Pancreatic pseudocysts are fluid collections that do not resolve, often communicative with the pancreatic ductal system, and slowly develop a circumferential capsule. They should be differentiated from the early extravasated fluid collections, having a dissimilar clinical significance and requiring a different therapeutic approach [1]. The species of pathogens isolated from an infected pancreas suggest an enteric origin in both pancreatic cyst and infected pancreatic necrosis. Nevertheless, the origin and route of the bacteria leading to infection of the pancreatic gland in AP are still unclear. Several mechanisms have been proposed to explain how these enteric bacteria reach the pancreas: translocation of bacteria from the gut, infection from the biliary tree or duodenum, as well as hematogenous or lymphatic spread from other sites. The most commonly isolated microorganisms in pancreatic infections are *E. coli*, *Enterococcus spp*., *Klebsiella pneumonidae *and, *Enterobacter spp*.; less frequent are *Staphylococcus spp*., *Pseudomonas aeruginosa*, *Streptococcus spp*., and *Bacteroides *[2].

Members of genus *Citrobacter *are Gram-negative, non-spore-forming rods belonging to the family *Enterobacteriaceae *and, as the name suggests, usually utilize citrate as a sole carbon source. These facultative anaerobes typically are motile by means of peritrichous flagella. They ferment glucose and other carbohydrates with the productions of acid and gas. They are oxidase negative, catalase and methyl red positive, Voges-Proskauer negative, and do not decarboxylate lysine. They are differentiated by their ability to convert tryptophan to indole. Of the dozen species, *C. freundii*, *C. diversus*, and *C. amalonaticus *are linked to human disease [3]. Acute necrotizing pancreatitis associated with *Citrobacter *infections is rare, and up to now, few cases have been reported in the literature.

## Case presentation

**Figure 1:**
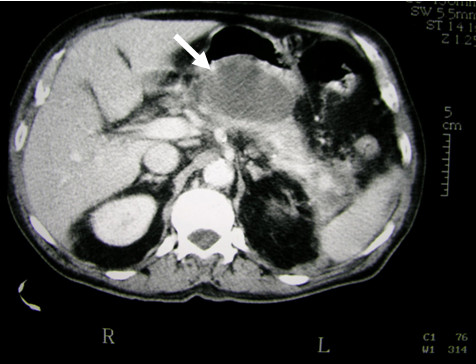
**Computed tomographic abdominal scan (CT) showing necrosis and presence of liquid in peripancreatic cavity**.

**Figure 2:**
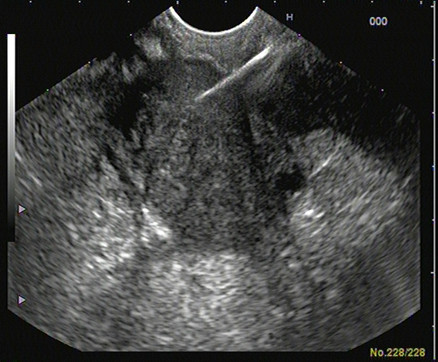
**Endoscopic ultrasound-guided fine needle aspiration of the pancreatic mass**.

A 80-year-old Caucasian man presented to our hospital with acute right lower quadrant and periumbilical abdominal pain. He had no history of previous alcohol abuse, cholelithiasis, abdominal trauma or surgery, nor ingestion of raw food or medications. On admission, hematological tests revealed normal hematocrit and platelet counts, and an increased white blood cell count of 23,800 mm^-3 ^(reference range: 4,000 to 10,000 mm^-3^) with 37.8% lymphocytes. The biochemical test results were within the reference range: ALT: 11 U/L (0 to 35), AST: 12 (0 to 35), glucose: 123, urea: 49, and creatinine: 1,1, Na: 142, K: 4.9, and serum amylase: 3157 UI/L (10 to 110). A computed tomographic (CT) abdominal scan was performed revealing necrosis over 30% of the pancreas and the presence of liquid in the peripancreatic cavity (Figure 1). With this finding, it was decided to perform an endoscopic ultrasound. The pancreatic parenchyma showed a slightly abnormally structured and irregular mass-like aspect on the head, compatible with an inflammatory process. The pancreatic body reflected a homogeneous pattern with an irregular and slightly dilated main pancreatic duct. In the neck of the pancreas, a cystic lesion with a dense aspect of 42.2 × 35.1 mm was revealed, probably related to a pseudocystic versus postnecrotic cavity. A gallbladder with hyperechogenic foci without an acoustic shadow floating inside, which is compatible with microlithiasis, was described. Endoscopy ultrasound fine needle aspiration (EUS-FNA; by using lineal equipment and a 22G needle puncture) was performed over the injury in the pancreatic head (Figure 2). Samples obtained were submitted for cytohistological and microbiological evaluation. Pathology results showed small fragments of pancreatic parenchyma without evidence of malignancy surrounded by areas of necrosis and inflammation. Microbiological analysis reveals a monoculture isolating *Citrobacter freundii *by biochemical testing with an API 20E system (API Biomerieux SA, Marcy I'Etoile, France yielded the numerical code 1604572 for the isolate. According to the API 20E database, this represented a "very good identification" for *C. freundii*. An analysis using *16SrRNA *gene by PCR-Sequencing method was performed to confirm the pathogen identity. *16SrRNA *gene of strain and *Citrobacter freundii *(FN997639) has the nearest kinship and are located in the same phylogenetic tree.

**Figure 3:**
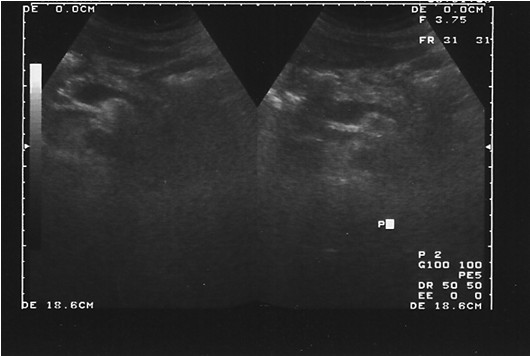
**Abdominal ultrasound showing the normal pancreas six months after the episode**.

Experiments with this bacterium in cell lines (cytopathic effect) and rats (i.p. injection) were performed and showed vacuolization of the cells as well as development of acute pancreatitis in the rats, demonstrating high levels of virulence of the strain. Susceptibility testing showed intermediate susceptibility to cefuroxim, although it was completely susceptible to ciprofloxacin. Oral ciprofloxacine (500 mg × 2) was administrated over six weeks. During treatment our patient progressed satisfactorily. A week after starting the treatment, he felt well and the abdominal pain gradually decreased. With the diagnosis of an acute necrotizing pancreatitis complicated with a pseudocystic percutaneous, he was discharged and referred to our Pancreatobiliary Unit. An abdominal ultrasound (Figure 3) performed six months later revealed a complete resolution of the previous inflammatory process.

## Discussion

In this study, *Citrobacter freundii *was detected in a sample obtained by EUS-FNA of a patient with pancreatic pseudocyst after an acute necrotizing pancreatitis.

EUS-FNA has emerged as an excellent tool to both image and sample pancreatic lesions [1]. It is considered the most sensitive and specific method of identifying pancreatic masses. The American Joint Commission on Cancer recommends EUS-FNA as the preferred diagnostic modality for pancreatic masses [2]. The presence of an on-site cytologist for immediate interpretation is a common practice in most high volume EUS centers [3]. On-site cytologic evaluation has been shown to increase the diagnostic yield by 10 to 15% [4-6] and can decrease procedure time and potential complications through avoidance of unnecessary needle passes once diagnostic tissue is obtained.

Most pathogens in pancreatic infection are gastrointestinal Gram negative bacteria; the colon seems to be the main source of pancreatitis related infections. Therefore, it is possible that bacterial translocation (BT) is the most important mechanism for contamination of pancreatic necrosis.

Pancreatic pseudocysts are more frequently polymicrobial (57%) than monomicrobial (43%). This fact contrasts with infected necrosis, where monomicrobial infections are usually found. Up to now anaerobes and fungi have rarely been reported; however, the bacterial spectrum may change in the near future due to the use of specific antibiotics leading to an increase in different microorganisms, especially fungi [2].

The diagnosis of pancreatic pseudocyst is based on clinical suspicion, imaging techniques, and demonstration of infection. Since clinical presentation may be very variable, pancreatic infection should be suspected in any patient with fever or suggestive signs or symptoms of sepsis within the context of AP. Once pancreatic pseudocysts have been diagnosed the treatment is complete drainage. Pancreatic pseudocysts do not resolve spontaneously and, if untreated, the prognosis for a patient is almost invariably death. Currently, two different approaches can be considered for primary drainage of a pancreatic pseudocyst: surgical and percutaneous.

Appropriate antibiotic therapy depends on the identification of the causative microorganisms and sensitivity testing. Meanwhile, several options have been recommended: a combination of ceftazidime and clindamycin; a combination of ciprofloxacin and metronidazole; or carbapenems as a single agent due to its extremely broad spectrum of activity [2].

Several trials have assessed the frequency of bacterial infection of necrotic areas in the natural course of severe AP without antibiotic intervention [7]. Results indicated an overall contamination rate of 24% within the first week of the onset of AP, increasing to 46 and 71%, respectively, in the second and third week.

*Escherichia fergusonii *was isolated from pancreatic carcinoma and cholangiosepsis [8] in a patient with a history of weight loss, jaundice, and acholic stools. After one day, *E. fergusonii *was grown as a single organism from the gallbladder fluid as well as from blood culture. Many other bacteria have been involved in AP [9]. A case of AP associated with brucellosis was reported in a 56-year-old patient with a seven-day history of fever, generalized myalgia and arthralgia, lower back pain, anorexia, and sweating. He experienced a sudden onset of abdominal pain accompanied by nausea and vomiting. He lived in an area of northwestern Greece where brucellosis is endemic. The CT scan revealed the presence of mild swelling of the pancreas without additional abnormality. The *Brucella *agglutinins were present in a titer and the blood culture grew *B. maletiensis *after eight days of incubation.

Also, *Mycobacterium tuberculosis *was isolated from a woman with a pancreatic mass. She was subsequently diagnosed with pancreatic tuberculosis via EUS-FNA. Intraprocedural immediate cytologic evaluation prompted samples to be sent for appropriate microbiological culture [10].

Studies evaluating the presence of bactDNA in biological fluids of patients with AP and other pathologies have rarely been reported in the literature. Madaria, *et al. *[11] reported the presence of *Citrobacter freundii *DNA and other pathogens (*Pseudomona aeruginosa *and *E. coli*) in four blood samples from patients with pancreatitis in Spain. There is no information in the literature regarding the pathogenic role of *C. freundii *in the development of infections in patients with AP; this bacterium has been shown to translocate in different experimental and clinical situations and is related to both biliary and intra-abdominal infections [12-14].

Enteric bacterial pathogens including *Vibrio cholerae *and enterotoxigenic *Escheriachia coli *often produce ADP-ribosylating enterotoxins that are mainly responsible for diarrheal diseases. Two well-characterized enterotoxins, cholera toxins produced by toxigenic strains of *Vibrio cholerae *and the heat-labile toxin (LT) produced by enterotoxigenic *E. coli *(ETEC) strains, have been detected to be virulence-like factors in *Citrobacter freundii *using immunological methods and PCR [15-17]. This suggests a possible gene transfer between *C. freundii *and this species [18].

One of the virulence factors attributed to *Citrobacter *spp. is the activation of transcription factors, kappa beta (NFκβ), concretely in *C. rodentium *(previously *C. freundii *biotype 4280). The NFκβ is a nuclear transcription which regulates the expression of a large number of genes that are critical for the regulation of apoptosis, viral replication, tumorigenesis, inflammation, and various autoimmune diseases. The NFκβ can be activated via different pathways. The most common, called the classical pathway, is triggered in response to microbial or pro-inflammatory cytokine injury, leading to a recruitment and activation of the Iκβ-kinase (IκK) complex which includes the scaffold protein NFκβ essential modulator, NEMO, also named, IκKγ [19-21].

It is possible that in pancreatic infections, NFκβ may have an additional role to perform, for example, in the expression and production of pro-inflammatory cytoquines during *C. freundii *infection and possibly in bacterial clearance.

## Conclusion

The patient's progress has been favorable. Final diagnosis isolated *C. freundii *based on a positive culture of an EUS-FNA sample. On-site cytology feedback was crucial to the successful outcome of this case as immediate interpretation of the FNA sample directed us to the appropriate cultures and, ultimately, the curative therapy. To our knowledge, this is the first reported case of isolated pancreatic *C. freundii *diagnosed by EUS-FNA.

## Abbreviations

AP: acute pancreatitis; BT: bacterial translocation; CT: computed tomography; ETEC: enterotoxigenic *E. coli; *EUS-FNA: endoscopy ultrasound fine needle aspiration; LT: labile toxin.

## Consent

Written informed consent was obtained from the patient for publication of this case report and accompanying images. A copy of the written consent is available for review by the Editor-in-Chief of this journal.

## Competing interests

The authors declare that they have no competing interests.

## Authors' contributions

ALL performed the tissue sample processing and microbiological analysis and was a major contributor in writing the manuscript. JLN, JIC, JIG and EDM performed the endoscopy and prescribed treatment, and followed the patient's progress during hospitalization. All authors read and approved the final manuscript.
